# Preferential hand use by captive chimpanzees (*Pan troglodytes*) in manual and tool digging

**DOI:** 10.1007/s10329-019-00727-y

**Published:** 2019-04-20

**Authors:** Alba Motes-Rodrigo, R. Adriana Hernandez-Aguilar, Matthias Laska

**Affiliations:** 1 IFM Biology, Linköping University, 581 83 Linköping, Sweden; 2 Department of Early Prehistory and Quaternary Ecology, University of Tübingen, Tübingen, Germany; 3 Department of Biosciences, Centre for Ecological and Evolutionary Synthesis (CEES), University of Oslo, Blindern, Oslo, Norway

**Keywords:** Chimpanzees, Hand preferences, Digging, Tool use, *Pan troglodytes*

## Abstract

Digging for underground storage organs of plants has been reported in various populations of wild chimpanzees (*Pan troglodytes*). However, it is unknown so far whether chimpanzees display lateral biases in manual digging as direct observations of this behavior are still lacking. It was therefore the aim of the present study to assess, for the first time, hand preferences for digging in a group of nine captive chimpanzees. We found that with only one exception, all individuals engaged in manual digging for buried food. Five individuals displayed a significant right-hand preference, two a significant left-hand preference, and one was ambidextrous. No apparent differences between males and females were found with regard to the direction or strength of hand preferences for manual digging. Only one out of four parent–offspring pairs was congruent in their preferred hand for manual digging. Three of the eight chimpanzees who dug manually also used tools in order to excavate buried food. Among those three individuals, one displayed a significant right-, one a significant left-hand preference, and one was ambidextrous. Only one of these three chimpanzees was consistent in preferring the same hand for manual and tool digging. The present findings are in line with the notion that chimpanzees display significant hand preferences at the individual level for haptic-guided behaviors, with a tendency for the right hand.

## Introduction

Chimpanzees in the wild are known to exploit food sources that require either manual processing or the use of tools in order to access and/or process them. This includes, but is not restricted to, the cracking of nuts using stones as hammers (e.g., Sakura and Matsuzawa 1991), the extraction of termites from underground nests (e.g., Sanz et al. 2004), and the extraction of honey from both arboreal beehives and underground nests (e.g., Boesch et al. 2009; Estienne et al. 2017). Only one other primate species is known so far to use tools to access underground foods: the bearded capuchin (*Sapajus libidinosus*; Mannu and Ottoni 2009; Falótico et al. 2017). Hand preferences are individual biases in the use of one hand on a single task, measure, or dependent variable (McGrew and Marchant 1997; MacNeilage et al. 1987). Several studies reported that chimpanzees display significant hand preferences in such tasks at the individual level (nut-cracking: Sugiyama et al. 1993; Humle and Matsuzawa 2009; termite fishing: McGrew and Marchant 1992; Sanz et al. 2016).

Wild chimpanzees have also been reported to excavate underground storage organs of plants (McGrew and Marchant 1997; Hernandez-Aguilar et al. 2007; Hockings et al. 2010; Lanjouw 2002). This digging behavior is particularly interesting from an evolutionary point of view, as it has been hypothesized that underground storage organs were used by early hominins as fallback foods in the initial colonization of savanna habitats (Laden and Wrangham 2005; Wrangham et al. 1999). The study of this behavior can thus provide valuable insights into the behavioral adaptations that hominins underwent when transitioning from the rainforest to the savanna. Furthermore, foraging for and consuming underground storage organs of plants is also a behavior currently present in hunter-gatherer populations (Lee 1979; Vincent 1985), which provides an evolutionary framework for the study of foraging for underground storage food sources. Chimpanzees are a suitable candidate species to create and test hypotheses concerning early hominin behavior (Pickering and Domínguez-Rodrigo 2010, 2012), as they are our closest living relatives (together with bonobos, *Pan paniscus*) and share dry and seasonal environments similar to those reconstructed for early hominins (Hernandez-Aguilar et al. 2007).

Digging for underground storage organs has been reported in different populations of chimpanzees (McGrew and Marchant 1997; Hernandez-Aguilar et al. 2007; Lanjouw 2002; Gaspersic and Pruetz 2011), but the occurrence of preferential hand use in this behavior has never been assessed until now. In a similar context, the obtention of water from dry riverbeds, two studies so far have investigated the laterality of digging and concluded that chimpanzees were ambidextrous for this behavior (McGrew et al. 2007, 2013). This conclusion was based on etho-archaeological evidence—a lack of significant differences in the volume of sand piles on the left and right sides of abandoned wells dug by chimpanzees. Although etho-archaeology is a valuable tool to study the past behavior of a species, it only allows to infer potential behaviors based on indirect evidence. In order to validate hypotheses based on etho-archaeological data, experimental approaches that provide direct evidence are necessary. Consequently, we designed an experiment that simulated digging for underground buried foods in order to assess laterality biases for this behavior in chimpanzees, which would allow us to replicate the findings of McGrew et al. using observational data. Digging in chimpanzees occurs in two modalities: manually (Lanjouw 2002; McGrew et al. 2007) and using tools (Hernandez-Aguilar et al. 2007; Gaspersic and Pruetz 2011). This duality allows for investigating the effect that tool use may have on the direction and strength of hand preference in digging for underground food. It was therefore the aim of the present study to assess, for the first time, individual hand preferences in manual and tool digging in captive chimpanzees. As our study population included several parent–offspring pairs, this allowed us to additionally assess whether individual hand preferences for digging were consistent within pairs.

## Materials and methods

### Ethical note

The experiments reported here comply with the *Guide for the Care and Use of Laboratory Animals* (8th edition, National Research Council, 2011) and also with the *American Society of Primatologists’ Principles for the Ethical Treatment of Primates*. A special ethical approval was not required because all experiments are considered as environmental enrichment for the animals and are thus covered by current Norwegian and Swedish Animal Welfare Laws for animals kept in zoos.

### Subjects

A group of nine chimpanzees (*Pan troglodytes*) kept at the Kristiansand Zoo (Kristiansand, Norway) was studied. The group included four males and five females whose demographic data are listed in Table 1.

**Table 1:** Demographic data of the nine chimpanzees included in the present study

Name	Sex	Year of birth	Age class	Origin	Parents	Offspring
Dixi	F	1977	Adult	Munich Zoo	–	Jane, Tobias
Julius	M	1979	Adult	Kristiansand Zoo	–	Junior, Yr
Josefine	F	1983	Adult	Öland Zoo	–	
Miff	F	1987	Adult	Copenhagen Zoo	–	Knerten
Tobias	M	1994	Adult	Kristiansand Zoo	Dixi	–
Jane	F	1999	Adult	Kristiansand Zoo	Dixi	Yr
Knerten	M	2000	Adult	Kristiansand Zoo	Miff	–
Junior	M	2003	Adult	Kristiansand Zoo	Binni, Julius	–
Yr	F	2011	Infant	Kristiansand Zoo	Jane, Julius	–

The chimpanzee enclosure at Kristiansand Zoo comprised an indoor and an outdoor exhibit plus the dormitories off-exhibit. The outdoor exhibit where the experiments reported here took place was a semi-naturally forested island of 1840 m^2^ surrounded by a water moat. The study took place in a 28-m^2^ area located at the southeast side of the outdoor exhibit composed of natural soil and vegetation. The chimpanzees had free access to the outdoor exhibit during the day and were fed before being released to the outdoor exhibit in the morning (ca. 09:00 h). Midday feeding took place at the indoor enclosure, whereas midafternoon feeding occurred in the dormitories. The diet of the chimpanzees was mainly composed of vegetables and fruits, complemented with primate pellets, nuts, honey, small quantities of meat, seeds, and yogurt. In addition, parts of plants (leaves and bark) occurring naturally in the outdoor exhibit were also consumed by the chimpanzees. Water was available ad libitum in both the indoor and outdoor enclosure.

### Experimental design

**Fig. 1:**
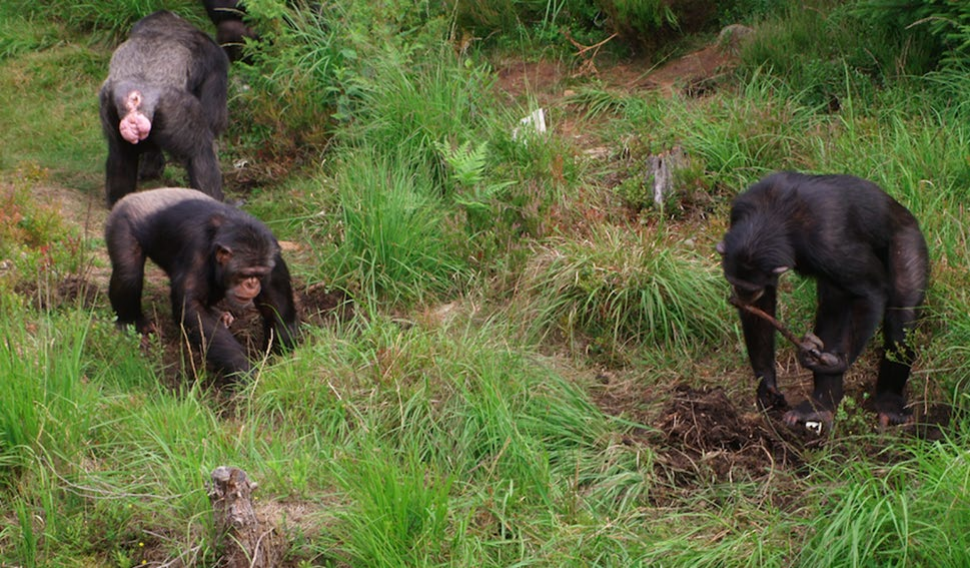
Chimpanzees performing the digging task. All chimpanzees performed this task adopting a quadrupedal position

Four holes were dug using gardening spades in the study area of the outdoor exhibit, keeping a minimum distance of 1.5 m between the holes to allow for the simultaneous performance of digging behaviors in different holes by different individuals (Fig. 1). The holes were 15 cm wide and 30 cm deep, in an attempt to emulate the dimensions of the holes excavated by wild chimpanzees when obtaining underground storage organs of plants described by Hernandez-Aguilar et al. (2007).

The habituation of the chimpanzees to the set-up was conducted during 3 days when the holes were left open with a piece of fruit inside (grape, cherry, or plum) and were marked with a yellow flag at the edge of the opening consisting of a wooden bamboo skewer (dimensions: 30 cm × 0.3 cm, weight: 2 g) and a yellow post-it sticker. This marking technique was kept throughout the study. During the testing period (75 days) the piece of fruit placed inside the hole was covered with soil to the brim and compacted manually and by foot.

The study site was prepared every day prior to the time when the chimpanzees were released into the outdoor exhibit (approximately at 08:00 h) by reshaping the holes to their original dimensions, placing the fruit inside at a depth of 30 cm and marking each hole with a flag.

All occurrences of digging during the testing phase were recorded using a digital video camera (Canon Legria HF M56) during approximately 3 h, starting when the chimpanzees accessed the outdoor exhibit between 08:00 and 09:00 a.m. A digging bout was defined as the period of time during which an individual was continuously digging (McGrew and Marchant 1992), starting at the moment the chimpanzee introduced a hand or a tool in one of the holes and ending when the subject abandoned the hole. For our analyses, only the first hand use from each bout was included (Braccini et al. 2010). These hand uses could be manual or involving a tool. We chose this data collection method (“bout-wise” only considering the first hand use of each bout) instead of recording each individual response or event during a bout (“event-wise”) in an attempt of recording strictly independent behavioral data points and to prevent pseudo-replication (Marchant and McGrew 2013).

### Data analysis

Only individuals for which at least five bouts were recorded, were included in the analyses. To determine the *direction* of individual hand preferences, binomial *z*-scores were calculated to classify the individuals as showing a right (*z* ≥ 1.96), left (*z* ≤ − 1.96), or no hand preference (− 1.96 < *z *<1.96). The absolute values of the *z*-scores were used to assess the *strength* of hand preference. In order to compare our results with previous studies that did not use *z*-scores, we also calculated the Handedness Index (HI) scores using the formula (*R*− *L*)/(*R* + *L*) to additionally report the direction of hand preference. In the calculation of the HI index, *R* and *L* are the number of right-hand and left-hand uses, respectively. HI-scores range from − 1 to +1, with negative values reflecting left-hand preferences and positive values reflecting right-hand preferences (Hopkins 1994). The absolute value of HI-scores, which ranges from 0 to 1, was also calculated to compare the strength of hand preference with previous studies that employed this measure. The height of the value of the absolute HI-score reflects how strong the hand preference is (Hopkins 1994). We did not perform statistical tests at the group level due to the small sample size of our study population. Sexes were only descriptively compared as we only included four males and four females in the study.

Data were analyzed using R (R Core team, 2014), and the alpha level was set at 0.05. All tests were two-tailed.

## Results

### Hand preference in manual digging

We recorded a total of 740 manual digging bouts. With only one exception (the oldest female, Dixi), all chimpanzees engaged in manual digging for hidden food. Details about the manual and tool digging techniques observed are described in a manuscript currently in press (Motes-Rodrigo et al. in press). Based on their *z*-scores, out of the eight individuals who were observed manually digging, five (Junior, Knerten, Tobias, Miff, Yr) displayed a significant right-hand preference, two (Jane and Julius) a significant left-hand preference, and one (Josefine) was ambidextrous (Table 2). Although the low number of individuals prevented any statistical comparisons between sexes, it should be mentioned that no apparent differences between males and females were found with regard to the direction of hand preferences: among males, three individuals were right- and one was left-hand-preferent, and among females, two individuals were right- and one was left-hand-preferent, and one was ambidextrous. Similarly, there were no apparent differences in the strength of hand preferences between sexes when mean absolute HI-scores were compared, with males (*N* = 4) and females (*N* = 4) having mean absolute HI-scores of 0.77 (range, 0.60–0.95) and 0.53 (range, 0.07–0.75), respectively. When mean absolute *z*-scores were compared between sexes, males (7.44, range, 12.19–4.32) showed stronger hand preferences than females (3.87, range, 7.72–0.93). However, when the ranges of mean absolute HI and *z*-scores were compared between males and females, females’ ranges were much broader than males’ ranges, suggesting that this disparity between results is the consequence of our small sample size.

**Table 2:** Manual digging

Subject	Sex	Left hand	Right hand	HI-score	Hand preference	*p* value	*z*-score
Julius	M	162	4	− 0.95	L	< 0.001	− 12.186
Junior	M	11	44	0.60	R	< 0.001	4.315
Jane	F	31	5	− 0.72	L	< 0.001	− 4.167
Knerten	M	8	75	0.81	R	< 0.001	7.244
Tobias	M	10	62	0.72	R	< 0.001	6.010
Yr	F	14	96	0.75	R	< 0.001	7.723
Josefine	F	90	104	0.07	A	0.351	0.933
Miff	F	5	19	0.58	R	0.007	2.654

*L* refers to a significant left-hand preference, *R* to a significant right-hand preference, *A* to an individual being ambidextrous

Only one out of the four parent–offspring pairs in the chimpanzee group was congruent in their preferred hand for manual digging (Miff and her son Knerten both significantly preferred their right hand in this task) whereas the other three parent–offspring pairs (Julius and his son Junior, Julius and his daughter Yr, and Jane and her daughter Yr) were not congruent (with one individual of a dyad significantly preferring its right hand and the other individual significantly preferring its left hand).

### Hand preference in tool digging

We recorded a total of 71 tool-digging bouts. Only three of the chimpanzees were observed to spontaneously dig with tools in order to dig for hidden food. Among those three individuals, one (Josefine) displayed a significant right-hand preference, one (Julius) a significant left-hand preference, and one (Junior) was ambidextrous (Table 3). Julius was consistent in his left-hand preference for both manual and tool digging, while Josefine and Junior showed a significant right-hand preference for one of the digging modalities but were ambidextrous for the other digging modality. There were no apparent differences in the strength of hand preference concerning the digging technique, with mean absolute HI-scores of 0.65 for both manual digging and tool digging, respectively. However, when mean absolute *z*-scores were compared between digging techniques, manual digging was found to elicit stronger hand preferences (5.65, *N* = 8) than tool digging (1.92, *N* = 3). The tools that the chimpanzees employed for digging included sticks and twigs, one long pine cone, and a PVC tube, which they transported into the study area from other parts of the outdoor exhibit or, in a few cases, even from the indoor exhibit. Additionally, the chimpanzees were observed to use as tools the bamboo skewers used in the experimental setup to indicate the position of the digging sites for excavating soil. Details on the digging tools used in this experiment are described in Motes-Rodrigo et al. (in press).

**Table 3:** Tool digging

Subject	Sex	Left hand	Right hand	HI-score	Hand preference	*p* value	*z*-score
Julius	M	12	2	− 0.71	L	0.013	− 2.405
Junior	M	2	7	0.56	A	0.18	1.333
Josefine	F	2	10	0.67	R	0.039	2.021

*L* refers to a significant left-hand preference, *R* to a significant right-hand preference, *A* to an individual being ambidextrous

## Discussion

To the best of our knowledge, the present study reports the first direct observations on preferential hand use in chimpanzees for digging. With only one exception, all individuals that dug manually displayed a significant hand preference and in the majority of cases this was a right-hand preference. Two prior studies (McGrew et al. 2007, 2013) inferred that wild chimpanzees in Semliki (Uganda) were ambidextrous when digging wells to obtain drinking water in dry riverbeds. However, these results were based on indirect evidence—the volume and weight of the sand piles accumulated at the right and left sides of abandoned wells dug by chimpanzees—rather than on direct observation. Furthermore, the authors assumed that each sand pile was created by the corresponding hand, meaning that the right hand was used to create the right pile and vice versa. However, in the present study, we observed that the chimpanzees also dug in a crossed fashion, with the right hand creating a pile on the left side and vice versa, and that several individuals dug successively in the same hole, taking turns, and thus more than one individual contributed to creating one pile of excavated soil. Therefore, the conclusion of McGrew et al. (2007, 2013) that chimpanzees are ambidextrous for digging should be revised in the light of the novel experimental data presented here. Our results exemplify that although etho-archaeological data are necessary and useful to formulate hypotheses regarding past behavior, it is necessary to test and validate these hypotheses using observational data, as behavior is often more flexible and variable than it can be inferred using indirect evidence.

Several authors have proposed that the characteristics of the foraging niche that a particular species exploits, may influence the motor patterns of the species. Terrestriality, for example, has been suggested to have played an important role in the development of primate technology (Meulman et al. 2012; Heldstab et al. 2016). Terrestriality also facilitates the access to underground food sources, a process mainly guided by the haptic rather than the visual sensory system. Other studies have found that the motor learning dimension of foraging may have been a driving force in the evolution of complex foraging niches (Schuppli et al. 2016). Our finding that the majority of individuals in our, admittedly, small study population displayed a right-hand preference in manual digging is in line with other studies on haptic-guided behaviors such as ant-dipping and palm heart extraction among the chimpanzees of Bossou in Guinea (Humle and Matsuzawa 2009). A higher incidence of right- than of left-hand preference has also been found in captive chimpanzees performing a haptic-guided task in which the individuals had to retrieve food from an opaque bucket filled with water (Lacreuse et al. 1999). In contrast to our results, studies of primates other than chimpanzees comparing visual and haptic-guided tasks found greater left-side biases in the latter type of task (McGrew and Marchant 1997), while another study did not find significant differences in the direction or strength of hand preferences between visual and haptic-guided tasks performed by spider monkeys (*Ateles geoffroyi*) (Laska 1996). Therefore, further research is needed to elucidate if there is a consistent effect of the predominant sensory modality on the strength or direction of preferential hand use and, if this is the case, how this effect may vary between species.

In our small study population, we failed to find apparent sex differences in the *direction* of preferential hand use in digging. Our results regarding apparent differences in the *strength* of hand preferences between sexes differed depending on whether absolute mean HI values or *z*-scores were considered. These differences obtained when using different statistical methods are likely to be an artifact of our small sample size. Sex differences in hand preferences have been found in wild chimpanzees bimanually manipulating fruits (Corp and Byrne 2004), wild chimpanzees termite fishing (Sanz et al. 2016) and captive chimpanzees performing a tool use task designed to mimic termite fishing (Hopkins et al. 2009). However, other studies found no significant differences between male and female chimpanzees in the preferred hand for reaching and grooming (Boesch 1991). Further studies should therefore assess whether sex differences in chimpanzee hand preferences exist and if they may be task-specific or derived from the statistical methods employed.

Our finding that only one out of four parent–offspring pairs were congruent in their preferred hand for digging does not seem to support the notion of heritability of hand preferences. Other studies in chimpanzees yielded mixed results with regard to genetic factors affecting preferential hand use. Whereas some studies reported that genetic relatedness had a significant effect on the preferred hand, e.g., in a task mimicking termite fishing (Hopkins et al. 2015), simple reaching (Hopkins et al. 1994) and tool use (Lonsdorf and Hopkins 2005), other studies failed to find such a genetic link, e.g., in the bimanual coordinated tube task (Hopkins 1999) or in nut-cracking (Boesch 1991). Here, too, further studies are needed to elucidate whether, or to what degree, a genetic influence on the expression of hand preferences may be task-specific. In addition to genetic determinants, the close contact between parent–offspring pairs may affect the congruency in hand preference between members of such a dyad. Hopkins et al. (2006) found stronger congruency in hand preference between chimpanzees and their mothers than between chimpanzees and their fathers. This result could indicate that (perhaps in addition to a maternal inheritance of hand preference) higher social association rates with mothers may affect hand preference. However, it is difficult to disentangle the genetic and non-genetic factors affecting hand preference, as evidenced by the lack of statistical difference in hand preferences between mother–offspring pairs reared together or apart (Hopkins et al. 2006). Therefore, although it is possible that observational learning may play a role in determining hand preferences, the nature of this influence remains unknown (Hopkins et al. 2015).

Finally, we found that only three of the eight chimpanzees who engaged in manual digging for buried food also used tools to excavate food hidden underground. At this point, it is difficult to decide which reasons may underlie this difference in the occurrence of manual and tool digging. One possibility is that the holes in our study were not deep enough or the substrate was not hard enough (Hernandez-Aguilar et al. 2007) to elicit the use of tools, as manual digging sufficed to obtain the buried foods in most cases. Similarly, differences in substrate characteristics could be at least partly responsible for the differences in hand preferences found between the present study and the results reported by McGrew et al. (2007, 2013). Further studies on chimpanzee digging behavior should explore the effect that variables related to the substrate (e.g., particle size, water content, hole depth, abundance of roots and stones) may have on the frequency of tool use and chimpanzee hand preferences for digging. Alternatively, inter-individual differences in previous experience with tool use in other contexts, or a lack thereof (Sanz and Morgan 2013), may also account for the low frequency of tool digging found in our study. Similarly, inter-individual differences in physical strength may contribute to an animal’s decision to use or not to use tools for digging, possibly depending on the compactness of the soil. The discovery and acquisition of efficient tool use in a novel context may, of course, take time. Whereas the learning of tool use in a task simulating termite fishing has been reported to take captive chimpanzees only a few days (Paquette 1992), we cannot exclude the possibility that the 75 days that we observed our study population in the digging task may have been too short to allow all individuals to acquire this skill. Whether observational learning, which has been suggested to affect the acquisition of tool use in certain motor tasks in chimpanzees (Price et al. 2009), may have contributed to our finding that only a fraction of our study population used tools for digging warrants further investigation.
